# Will applications on smartphones allow a generalization of telemedicine?

**DOI:** 10.1186/s12911-020-1036-0

**Published:** 2020-02-11

**Authors:** F. A. Allaert, L. Legrand, N. Abdoul Carime, C. Quantin

**Affiliations:** 1 Chaire d’évaluation Médicale des Allégations de Santé BSB et groupe CEN, Dijon, France; 2 Service de Biostatistiques et d’Information Médicale (DIM), CHRU Dijon; Univ. Bourgogne Franche-Comté, F-21000 Dijon, France; 3 Laboratoire ImViA, EA 7535, UFR des Sciences de Santé, Université de Bourgogne Franche-Comté, Besançon, France; 4 INSERM Clinical Investigation Center, clinical epidemiology/ clinical trials unit, CIC 1432 Dijon University Hospital Dijon, France; 5 Biostatistics, Biomathematics, Pharmacoepidemiology and Infectious Diseases (B2PHI), INSERM, UVSQ, Institut Pasteur, Université Paris-Saclay, Paris, France; 6 Service de Biostatistique et d’Informatique Médicale - BP 77908, CHU de Dijon, CEDEX, 21079 Dijon, France

**Keywords:** Telemedicine, Smartphones, Cost effectiveness

## Abstract

**Background:**

Telemedicine is one of the healthcare sectors that has developed the most in recent years. Currently, telemedicine is mostly used for patients who have difficulty attending medical consultations because of where they live (teleconsultation) or for specialist referrals when no specialist of a given discipline is locally available (telexpertise). However, the use of specific equipment (with dedicated cameras, screens, and computers) and the need for institutional infrastructure made the deployment and use of these systems expensive and rigid. Although many telemedicine systems have been tested, most have not generally gone beyond local projects.

Our hypothesis is that the use of smartphones will allow health care providers to overcome some of the limitations that we have exposed, thus allowing the generalization of telemedicine.

**Main body:**

This paper addresses the problem of telemedicine applications, the market of which is growing fast. Their development may completely transform the organization of healthcare systems, change the way patients are managed and revolutionize prevention. This new organization should facilitate the lives of both patients and doctors.

In this paper, we examine why telemedicine has failed for years to take its rightful place in many European healthcare systems although there was a real need. By developing the example of France, this article analyses the reasons most commonly put forth: the administrative and legal difficulties, and the lack of funding. We argue that the real reason telemedicine struggled to find its place was because the technology was not close enough to the patient.

**Conclusion:**

Finally, we explain how the development of smartphones and their current ubiquitousness should allow the generalization of telemedicine in France and on a global scale.

## Background

Telemedicine is one of the healthcare sectors that has developed the most in recent years. A recent study based on the analysis of telemedicine in seven European countries (Switzerland, the UK, the Netherlands, Spain, France, Italy and Belgium) has made it possible to compare the maturity of the telemedicine markets in Europe and in the USA, which is considered to be the most advanced in the field. This study confirmed that telemedicine was less developed in European countries than in the USA, but it also indicated that Europe was starting to close the gap [1].

The European Commission has started to address the current and potential impact of telemedicine applications, and has also begun promoting telemedicine in its 28 member states [2]. However, these states have been given no particular timeline for integrating telemedicine into their public health services [3].

Another recent study conducted among people aged 16 to 74 in the European member states showed that mobile phones were among the devices most used to access the internet [4]. The nearly ubiquitous use of smartphones may therefore be a valuable means for European countries that are eager to develop the use of telemedicine.

For a number of years already, telemedicine has been heralded as the future of the medical practice for the twenty-first century. And yet, it is still far from the routine, except in countries like Canada [5] or Norway [6] where distance and low population density have made telemedicine indispensable. Today, telemedicine is a way to bridge the accessibility gap, compensating for the lack of medical centers in rural zones and the increase in the elderly population. Yet most telemedicine systems remain experimental and have never been used on a large scale. Through the analysis of the development of telemedicine in France, which mirrors the experience in many other European countries, we suggest that the specially-designed telemedicine equipment is in fact responsible for the limited growth of the service, because this equipment has never been close enough to the end user. We argue that smartphones are the “missing link” in telemedicine. Sooner or later, smartphones and their applications will be the real service provider for telemedicine, replacing the current equipment which is too big, too expensive and already technologically obsolete.

In France, for nearly 25 years, telemedicine has been the focus of the reflection and the center of numerous projects put forth public health officials. In 1996, we carried out an experiment in image transmission between the Dijon Faculty of Medicine and Harvard to demonstrate the relevance of telemedicine for pathology, a field that seemed straightforward and effective at the time [7]. Since then, a large number of telemedicine systems have been developed, in the world of medical imaging in particular, but the systems behind these concrete achievements have nevertheless struggled to gain traction. Eight years ago, the French law concerning health services marked a turning point and raised the hopes of many. It provided a definition of telemedicine and created the long-awaited legal framework for this practice, which could take the form of teleconsultation, tele-expertise, remote medical monitoring, remote medical assistance or even correspond to the “medical response that is provided as part of medical regulation”.

At the same time, the European texts also gave telemedicine official recognition by defining it as “the provision of healthcare services, through the use of information communication technology (ICT), in situations where the health professional and the patient (or two health professionals) are not in the same location. It involves secure transmission of medical data and information, through text, sound, images or other forms needed for the prevention, diagnosis, treatment and follow-up of patients” [8].

Despite legal recognition, telemedicine has made only limited progress in certain privileged sectors mainly involving images as stated above, or in remote areas or areas with little medical access where even a service that seems “artisanal” is better than nothing. A recent review of the legal framework of telemedicine shows that, at the European level, many issues such as medical liability and of medical *leges artis* still lack uniform regulation, and these gaps may jeopardize the growth of an internal European health services market and hamper the development of telemedicine in the European zone [9].

There are two regularly mentioned obstacles to the development of telemedecine. The first is the difficulty of defining a recognized act of telemedicine that would be covered by health insurance without resulting in inconsistencies, in competition prohibited by the code of medical ethics or in the artificial duplication of acts. On September 15th, 2018, the price was set for an act of teleconsultation, yet coverage is still very limited both in terms of territory (areas that lack medical facilities), field of use (nursing homes) and the number of authorized acts per year (Ministerial Order dated August 16th, 2018). This publicly decreed implementation, which is to be accompanied by an equipment package, could potentially accelerate the development of telemedicine, especially if it could be extended to other fields. However, as we intend to demonstrate, this important step in favor of telemedicine may have little effect. The approach is similar to that carried out in the US with the promulgation of the Bipartisan Budget Act of 2018 [10], which marked a considerable advance by expanding the coverage of many telemedicine services so that Medicare Advantage plans could include delivery of telehealth services in a plan’s basic benefits. It also gave Accountable Care Organizations the ability to expand the use of telehealth services. The second obstacle is the issue of responsibility in the event of injury to a patient during a telemedicine procedure. Here again, there has been ongoing discussion since the very beginning of telemedicine, and these deliberations continue today [11].

Despite these efforts, the use of telemedicine in the form of teleconsultation, whose purpose is *“to enable a medical professional to carry out a remote consultation with a patient*” or in the form of a remote medical monitoring whose purpose is “*to enable a medical professional to interpret remotely the data necessary for the medical follow-up of a patient and, where appropriate, to make decisions relating to the management of this patient*” [12] has remained relatively stagnant.

As mentioned above, the technical conditions and the framework of responsibilities and remunerations continue to block the development of telemedicine. For example, the French government has recently decided to promote the development of telemedicine via financial incentives: national health insurance has financed a teleconsultation act at the same price as a regular consultation since September 15th, 2018. Yet the number of telemedicine acts remains underwhelming: 1 year after the reform, only 60,000 acts were recorded, while the government’s objective was 500,000.

In this paper, we discuss why telemedicine has failed for years to take its rightful place in many European healthcare systems [13–16] although there was a genuine demand for this type of service. By developing the example of France, this article analyses the reasons most commonly put forth for this failure [17], which are the administrative and legal difficulties as well as the lack of funding. We demonstrate that the real reason was that the technology was not close enough to the patient to be truly effective.

Our purpose is to draw a hypothesis that the generalized use of smartphones will make it possible, both technically and in terms of regulation, to broaden the use of telemedicine practices. However, the use of smartphones creates a new set of risks that will need to be carefully managed [18–20].

### The reasons for the economic failure of the current organization of telemedicine

The implementation of telemedicine as it stands is twofold, requiring information-gathering equipment on one side and the installation of information analysis equipment for expertise or consultation on the other. In the place where we find the patient, the equipment is generally composed of measuring instruments, cameras or other input devices in order to collect patient data; this equipment is coupled with a computer device whose software can transmit patient data securely. In the place where the expertise takes place, a computer system (usually a computer server) is equipped with software capable of analyzing, processing and transmitting this data to another terminal used by the physician.

In addition to developing the required computer software prior to implementation, both sides required significant investment in hardware and human resources. On the analysis side, it was necessary to set up a workstation with high definition hardware for viewing and transmitting good quality images. It was relatively expensive and complex until recent years, and unfortunately most existing installations are already obsolete. There was little anticipation of the mass production of low-cost cameras and the growth of low-cost teleconferencing or video exchange systems that democratized these processes and drove prices downwards. In addition, these often complex operating platforms required properly trained technicians, specific maintenance and the availability of medical staff who had to be present either at all times or during scheduled time slots to provide the service [16, 17].

A second challenge was connecting to high speed data transfer networks, which were not very widespread at the time, and even less in areas with lower population density, which were precisely the areas suffering most from the lack of health care. In this respect the situation is improving steadily, but even today many regions are not equipped with fiber optics, and low bit rates are still frequent.

Finally, for years, telemedicine has been reduced to experimental hospital environments or relatively expensive inter-hospital cooperation, without being able to really respond to the ever-increasing needs in rural areas, including the private sector and establishments such as nursing homes. Indeed, telemedicine required practically the same equipment and the same technical and maintenance constraints in the facilities hosting the patients and in those analyzing the data. Due to their cost and complexity, these structures were mainly available in hospitals or clinics, facilitating inter-hospital cooperation, and making progress in certain cases, mainly in specialized sectors also affected by a scarcity of medical personnel. However, the growing needs of the greatest number of patients, especially ambulatory patients, were not addressed. They still had to make their appointments well in advance and go to or be transported to the hospital where the telemedicine station was situated, resulting in a new set of costs. In response to this situation, patients were often referred directly to private or hospital specialists instead of the existing telemedicine platforms, notwithstanding the distance and the additional costs involved. As a result, the volume of requests for telemedicine procedures has stagnated and hospitals, carrying out tele-expertise sessions without financial compensation, had little interest in developing them for the ambulatory sector. Ambulatory care structures could certainly have had recourse to such assistance, but were not inclined to bear the costs directly, due in particular to organizational difficulties and the lack of staff.

### An example of how smartphone-based telemedicine could be organized to overcome the main limitations

The main reason for the failure of telemedicine was inadequate (and/or flawed) technology**.** With previous technologies, telemedicine was a relative failure due to a lack of local access for the patient and because it was most often used for remote expert relationships between doctors than for real patient teleconsultations. There was a missing link between the telemedicine structure and the patient, but this missing link appeared in the form of the smartphone. In just a few years, smartphones have developed the power to acquire and transmit high-quality text, sound, images and video that, until recently, only complex and expensive technical platforms could provide. They are easy to use, accessible to all and maintenance is almost non-existent. Moreover, their use of 3G, then 4G, and soon 5G, as well as their ability to use Wi-Fi networks, allow them to transmit different types of information over practically the entire territory, despite the increasingly rare coverage “gaps” in the networks.

The second main limitation was concerns related to data security. With smartphones, the protection of medical confidentiality on networks can benefit from cryptographic techniques, and patient records can be exchanged on electronic platforms placed at approved health data hosts in order to constitute electronic evidence, duly authenticated and time-stamped in the event of a medicolegal challenge, which is not currently the case on most current telemedicine systems.

Finally, from a public health point of view, there will be major benefits for health economics. Compared to acquisition structures, smartphones are cheap and almost the whole population already has one, including healthcare providers - even if they do not necessarily wish to use their personal phones for professional purposes. Having a smartphone for the purpose of telemedicine is not an excessive expense and could even fall within the scope of the telemedicine packages provided by the health insurance system for its development. Thanks to this potential to acquire information, even in video form, directly at the patient’s bedside, teleconsultations can be requested not only by doctors but also other health professionals such as nurses, or even by patients themselves. There will also be improvements in patient quality of life. We can take, as an example, the case of an elderly person living in a nursing home with a chronic venous ulcer-type wound whose deterioration worries the staff on a Friday afternoon. Without access to the expertise of a dermatologist or angiologist (who does not travel for this type of problem), the decision is made to refer the patient to the hospital in case the wound continues to get worse at the weekend. This single decision is very costly: it will lead to an administrative discharge and entry of the patient, the use of round-trip medical transport, an outpatient consultation and undoubtedly a hospital stay because it is Friday evening and the earliest possible return to the nursing home is Monday. For an elderly person, often transported without much explanation, panicked at the idea of going to the hospital, placed in an uncomfortable stretcher for hours sometimes in a situation of spatial and temporal disorientation, this is a psychologically traumatic situation. The alternative is simple, inexpensive and comfortable. The worried nurse takes a picture with a smartphone, fills out a form, connects with a remote diagnostic platform and receives in return, within 2 h, a message about wound management. As a result, the treatment is implemented without delay. An expense of around 30 euros has just saved a few hundred euros in expenses and a lot of unnecessary stress for both the patient and the care staff in charge of organizing a transfer. There are a large number of published examples of smartphone-based telemedicine demonstrating both its clinical effectiveness and cost-effectiveness in fields as varied as geriatrics [21], psychiatry [22], neurology [23], dermatology [24], and cardiology [25].

### Challenges

As with any introduction of a new system of organization, it is important to consider the consequences for the general economy of the health system and any possible negative effects [26, 27].

The first challenge is of course that the simplicity of telemedicine, almost comparable to a simple telephone call from the patient’s bedside, may become so popular that it is used without careful consideration of the patient’s symptoms. This could result in a risk of increased consultations and therefore costs, potentially exceeding the savings it should generate. According the American Medical association, “*despite its promise, telemedicine is not an appropriate model of care for all medical conditions. For example, telemedicine is inappropriate for encounters when a hands-on physical examination is crucial or critical data can be gleaned only through direct physical contact. More broadly, telemedicine is not the preferred approach when the technology does not allow physicians to meet established clinical standards*” [28]*.*

The second is that the intensive use of telemedicine, paradoxically, could ultimately contribute to a decline in medical demographics. If specialists located in rural areas are deprived of a portion of their consultations, the economic profitability of their practice could be jeopardized, leading them to settle elsewhere. This risk should not be neglected because in many places, it is not so much the lack of specialist doctors that has led to their absence as the lack of clients in regions with a low population density. This worry also concerns other medical services. For example, the spread of telemedicine will likely contribute to a reduction in ambulance [29] transports, but if demand drops, profits will drop, leading to a progressive disappearance of the service provider. What happens when these services are still needed but the groups that provide them have vanished?

The third potential harmful effect is that if bedside teleconsultation becomes widespread in all areas of patient care, the patient will be deprived of direct contact with the doctor and in particular of the possibility of talking directly to him/her and undergoing a thorough clinical examination. This situation would lead to a dehumanization of medicine, all the more unacceptable since telemedicine must on the contrary provide a superior quality of patient care. This aspect has been described in a recent paper exploring how smart technologies may create a double-edged sword for patient safety and effective therapeutic relationships. The authors underlined the need for regulatory guidelines and better education regarding the benefits and risks of these devices for both healthcare providers and patients [30].

The risk of a surge in telemedicine has been anticipated by the legislation on the management of teleconsultations, which, as indicated above, will be limited to certain territories, certain structures (including nursing home), and a maximum number per patient per year. Some see this restriction as a limit to the development of telemedicine, but it appears to be an essential condition for balanced development and for maintaining the service it must provide to the population.

Finally, it should be stated that the practice of telemedicine, which has been made possible at the bedside through the use of the smartphone, must be considered as a means of supplementing of diagnostic and therapeutic needs, but not as a practice intended to replace medical consultation. This fundamental principle must be taken into consideration in order to anticipate the changing organization of our health system and the extraordinary progress made possible by smartphones and connected health objects. It is predicted that some devices will soon provide direct screening for heart rhythm disorders, valve abnormalities, behavioral disorders, blood pressure measurement without a cuff, remote control of insulin pumps based on blood glucose levels, etc. Today it is difficult to imagine the technical developments that will take place in the next 5 years when artificial intelligence techniques reach maturity, but they risk going far beyond where we currently stand. A single glance at the developments that have occurred in recent years is proof enough. Who would have thought, for example, that we could measure a person’s blood pressure by analysing the micro-colour variations detected in the capillaries of the forehead skin, developing new micro-sensors for physicochemical parameters? The ultimate step in telemedicine, where the caregiver or doctor, acting as an intermediary between the patient and the care platform, will finally be eliminated, should be studied from a medical, financial and ethical point of view, because it is swiftly becoming a reality. How will it be evaluated, who will benefit from it, how will it be managed? New issues are constantly emerging.

## Conclusion

Smartphone-based telemedicine, made possible by a permanently available local multimedia personal communication platform, should not be considered as a simple new technology but should be anticipated as the main force behind the reorganization of our health system. Well beyond the currently available services, sometimes still in their beginnings, smartphones bear the promise of fully transformed and dematerialized healthcare systems. After overcoming the need for an on-site doctor, the coupling of telemedicine and artificial intelligence techniques may even call into question the very need for a doctor in many situations, whether locally or remotely. What is certain is that in the years to come the medical profession will have to adapt and change its practices. The challenge will then be to know if dematerialization will go hand in hand with dehumanization or if, on the contrary, physicians relieved of repetitive tasks will refocus on what is increasingly being lost: the human relationship with the patient and the accompanying attention, discussion, understanding and compassion. Louis Pasteur once said “a little science takes us far from God but a lot brings us back”; we can imagine that if the limited use of technology has diminished human relations, a lot could allow, on the contrary, the re-humanization of the medical profession.

## Data Availability

Not applicable.
